# In Vitro Studies on the Influence of Meloxicam on Cytotoxic Activity Induced by Risedronate Sodium in Canine (D-17) and Human (U-2 OS) Osteosarcoma Cell Lines

**DOI:** 10.3390/ani11113135

**Published:** 2021-11-02

**Authors:** Dominik Poradowski, Izabela Janus, Aleksander Chrószcz, Bożena Obmińska-Mrukowicz

**Affiliations:** 1 Department of Animal Physiology and Biostructure, Division of Animal Anatomy, Faculty of Veterinary Medicine, Wroclaw University of Environmental and Life Science, Kożuchowska 1, 51-631 Wrocław, Poland; aleksander.chroszcz@upwr.edu.pl; 2 Department of Pathology, Division of Pathomorphology and Veterinary Forensics, Faculty of Veterinary Medicine, Wroclaw University of Environmental and Life Sciences, C. K. Norwida 31, 50-375 Wrocław, Poland; izabela.janus@upwr.edu.pl; 3 Department of Pharmacology and Toxicology, Division of Pharmacology, Faculty of Veterinary Medicine, Wroclaw University of Environmental and Life Science, C. K. Norwida 31, 50-375 Wrocław, Poland; bozena.obminska-mrukowicz@upwr.edu.pl

**Keywords:** osteosarcoma, human, canine, risedronate sodium, meloxicam, interaction

## Abstract

**Simple Summary:**

The aim of this in vitro study was to reveal the pharmacological interactions between meloxicam and risedronate sodium, used jointly to induce a cytotoxic effect in canine (D-17) and human (U-2 OS) osteosarcoma cell lines. Meloxicam, a non-steroidal anti-inflammatory drug, is capable of intensifying the cytotoxic activity of risedronate sodium routinely used in bone tissue metabolic diseases. The cell cultures were incubated, tested, and evaluated according to standard protocols. The study demonstrated a greater susceptibility of canine osteosarcoma cells in vitro to the investigated drug combination than the human. In both cases, meloxicam alone showed low cytotoxic activity against the tested cell lines, but the two compounds combined were synergic.

**Abstract:**

The study describes the cytotoxic effect against human and canine osteosarcoma (U-2 OS and D-17) cell lines induced by risedronate sodium and meloxicam per se and in combination. Both cell lines were prepared according to standard procedures for cell cultures studies. The cell viability was estimated in both cell lines treated with chosen concentrations of risedronate sodium and meloxicam. The apoptosis assessment was carried out using TUNEL (terminal deoxynucleotidyl transferase dUTP nick end labeling) assay. EC_50_ values, computed for risedronate sodium and meloxicam cytotoxicity, showed comparable effects against the canine OS cell line in similar concentration of both drugs. In case of human OS, the stronger cytotoxic effect of risedronate sodium was proved. The EC_50_ values for meloxicam in both cell lines were, statistically, significantly different (* *p* < 0.05). Moreover, the cytotoxic effect of a combined administration of meloxicam and risedronate sodium in doses 100 µg/mL, compared with the negative control showed statistically significant differences. The human OS cell line was more resistant to both compounds than the canine OS cell line. The apoptotic effect in canine and human osteosarcoma triggered by risedronate sodium and meloxicam was statistically significant (*p* < 0.05). The cytotoxic effect induced with 100 µg/mL of risedronate sodium proved statistically significant differences between both tested cell lines compared to negative control. The results obtained with 10 and 100 µg/mL of meloxicam were not statistically significant. The study showed the synergic mechanism of action of risedronate sodium and meloxicam, but the concentrations used in vitro will not be possible to achieve in in vivo. Therefore, our results serve as basis only to design future studies on the tissue level.

## 1. Introduction

Osteosarcoma (OS), a malignant primary bone tumor of mesenchymal origin with a highly diverse histopathological structure, constitutes a treatment challenge in veterinary and human medicine. OS is diagnosed in approximately 80–85% of dogs with bone tumors, which makes it the most common type of bone neoplasm [1]. However, when all types of canine tumors are considered, its prevalence is moderate. Typically, OS is diagnosed in adult dogs aged 2–15 years, with a mean of 7 years [2,3,4,5]. Representatives of the large and giant breeds are the most predisposed to this type of cancer [1,6]. Moreover, despite the animal’s sex, mixed breeds are more predisposed to OS [7].

In dogs, OS affects most frequently the appendicular skeleton. It must be stressed, the genetic factor plays an important role in OS etiology [8]. Other potential causes of OS occurrence are metallic implant placement, ionizing radiation, minor chronic micro trauma, post-orthopedic surgery bone infracts, and bone infections [9].

Considering histopathological structure, location, age of occurrence, and also predisposing factors, human and canine OS exhibit similar features [10,11,12,13]. In our study, a comparison of responses of both OS cell cultures (U-2 OS and D-17) to meloxicam and risedronate, alone and in combination, can bring the answer if both substances used (combined or separated) in chosen concentrations can increase the cytotoxic effect in human and canine OS cell lines.

The pharmacological effect of meloxicam administration consists of the subsequent mechanism of action, in which three isoform cyclooxygenases play a crucial role: COX-1, COX-2, and COX-3 as a biochemical pathway of the arachidonic acid. The aforementioned acid is subsequently transformed into prostaglandin G (PGG_2_); prostaglandin H (PGH_2_); and finally, prostaglandin D, E, and F, prostacyclin (PGI_2_), and thromboxane A_2_ (TRA_2_). COX-1 (constitutive form) is present in organs, in which prostnoids play the physiological function, i.e., decrease the secretion of hydrochloric acid (HCL) in the glandular stomach mucosa or cause luteolysis in the ovary. Moreover, prostaglandins influence the circulatory system, i.e., blood vessel dilatation and thrombocytes aggregation modulation. They also stimulate the mobility of spermiums and modulate the smooth muscles’ contraction. The synthesis of the second isoform of cyclooxygenase (COX-2), known as inductive form, is stimulated with the inflammatory process mediators (i.e., interleukin-1, interleukin-6, and TNF-α) from monocytes, macrophages, fibroblasts, osteocytes, and chondrocytes. The additional variant of COX-1 is COX-3, isolated from the central nervous system and its role is not fully known. Routinely used in therapy, non-steroidal anti-inflammatory drugs (NSAIDs) are capable of non-selective inhibition of both cyclooxygenase isoforms, preferential inhibition of COX-2, or even selective inhibition of COX-2 [14].

Meloxicam is an enolic acid derivative (oxicam) with strong anti-inflammatory, analgesic, and anti-pyretic effect, used both in humans and dogs. The mechanism of action is based on the preferential inhibition of COX-2 in dogs and moderate-selective inhibition of COX-2 in humans [14,15]. The latter has clinical importance, because it has been shown in MG-63 cell cultures that meloxicam has an inhibitory effect on OS tumor growth, invasiveness, and metastases by COX-2 dependent and independent pathways. The low concentration of COX-2 in human OS (HOS and U2-OS) is interpreted as the factor in reducing the apoptotic effect of meloxicam in the mentioned cell lines [16]. The comparison with the canine OS (D-17) cell line with human OS cells (U2-OS) shall test the hypothesis if the differences in the meloxicam mechanism of action influence the cytotoxic effect observed in both cell lines.

The risedronate sodium belongs to the third generation of bisphosphonates. It shows very strong affinity to the bone tissue. Although risedronate molecules do not penetrate the cell cytoplasm, they accumulate in the bone matrix, causing the inhibition of hydroxyapatite degradation and the decrease in osteoclasts activity (apoptosis caused by mevalonate pathway inhibition) [17].

Risedronate sodium is routinely used in Paget’s disease and osteoporosis. The literature lacks wider information about the risedronate sodium in vitro cytotoxicity. The cytostatic activity of risedronate sodium has been evaluated rarely and only clinically in OS therapy. Murayama et al. [18] studied the cytotoxic effect of risedronate sodium in chosen cell lines (including U-2 OS) and proved its EC_50_ to be above 100 µM. These results encouraged us to design our experiment with the doses of the aforementioned substance in the range of 0.15 and 300 µg/mL. Moreover, Murayama et al. [18] stated the dose-dependent number of apoptotic cells, which supported the choice of the doses we selected. The TNF-related apoptosis-inducing ligand (TRAIL) seems to be one of the most promising candidates for neoplasms therapy. In the case of OS, some tumors can be TRAIL-receptor resistant. Bisphosphonates inhibit the protein prenylation, important in cell physiology and survival. Studies by Moon et al. [19] showed that preliminary treatment with bisphosphonates induces mRNA and protein expression of the TRAIL receptor (DR5). Bisphosphonates are able to induce the protein unprenylation in TRAIL-resistant cells and significantly increase TRAIL-mediated apoptosis by cellular activation of caspase-3.

Bisphosphonates deeply influence the bone tissue metabolism. This caused its use not only in osteoporosis therapy, but also in oncology as osteoclasts-mediated bone diseases treatment. It is known that this group of drugs is also able to inhibit the growth of soft tissue tumors, like breast cancer, renal cell carcinoma, and prostate cancer [20]. The therapeutic use of bisphosphonates (alendronate sodium or pamidronate sodium) narrows the bone tissue osteolytic processes in OS, decreases cell proliferation and cell viability, and provides analgesic effects [21,22].

The treatment of human and canine OS follows a similar regimen. The routine treatment involves a surgery (amputation/resection of tumor tissues or limb-sparing surgery) combined with a post-surgical chemotherapy protocol; radiotherapy can also be part of the treatment [23]. Regardless of the protocol, therapy is often supplemented with non-steroidal anti-inflammatory drugs, such as carprofen, metamizole, or meloxicam. Their anti-inflammatory, analgesic, and antipyretic activity is accompanied frequently by the anti-cancer effect [24].

In summary, the combined actions and interactions between bisphosphonates and NSAIDs in anti-cancer therapy seems to be a new research challenge. Before introducing any compound for in vivo studies in vitro investigations, such as those chosen by us in form of cell cultures, studies are needed. Therefore, the aim of this study was to evaluate the potential cytotoxic activity of risedronate sodium alone and in combination with meloxicam in human and canine OS cell lines. Moreover, the comparison of results achieved for the use of risedronate sodium/meloxicam, both combined and separately, may help to answer whether the compounds have any synergic or additive effect.

## 2. Materials and Methods

### 2.1. Tested Substances and Cell Line Preparation 

Canine (D-17) and human (U-2 OS) osteosarcoma (ATCC, Manassas, VA, USA) cell lines in culture flasks with a bottom area of 25 cm^2^ were incubated under a constant 5% flow of CO_2_ at 37 °C. Eagle’s Minimum Essential Medium (EMEM) and McCoy’s 5A culture medium (ATCC, Manassas, VA, USA) were supplemented with 10% fetal bovine serum (FBS) (Sigma-Aldrich, Burlington, MA, USA), 4 nM L-glutamine (Sigma-Aldrich, Burlington, MA, USA), 100 U/mL penicillin, and 100 µg/mL streptomycin (Sigma-Aldrich, Taufkirchen, Germany).

Meloxicam and risedronate sodium (Sigma-Aldrich, Taufkirchen, Germany) were diluted in double-distilled water. The culture media were used to obtain the desired concentrations of the tested compounds, for meloxicam (mel) 100 and 10, for risedronate sodium (rd) 100, 10 and 1 µg/mL. The concentration ranges of the tested substances were based on their chemical characteristic of solubility in water, on the literature data, and their maximal concentration in serum. Higher doses of meloxicam and risedronate (above the routinely used therapeutic doses), were used to observe their potential synergistic activity in vitro. The negative control was non-treated cells, and the positive control was cells treated with doxorubicin at 0.5 µg/mL.

### 2.2. Cell Viability Assessment

Cells from D-17 and U-2 OS lines were placed onto sterile, 96-well cell culture plates (TPP, Trasadingen, Switzerland). After 24 h incubation, the culture medium was replaced. Then, the cells were incubated with the tested compounds alone or in combinations (Table 1), at 37 °C for 72 h under a constant flow of 5% CO_2_.

Further to assessing cellular viability, an attempt at determining the half maximal effective concentration (EC_50_) was made. Cell viability was measured with MTT assay (Sigma-Aldrich, Burlington, MA, USA).

Four independent repetitions with each tested compound and its combinations were performed. The results are expressed as a mean value of those repetitions. EC_50_ was calculated only for meloxicam and risedronate sodium alone, while for combinations of the tested compounds, only mean values are provided.

### 2.3. Apoptosis Assessment with TUNEL Method

After the cell viability assessment, the concentrations of the tested compounds for which EC_50_ exceeded 50% were chosen for further investigations. This allowed us to investigate their pharmacological interactions and to perform the statistical analysis of the archived results.

The cells from the canine and human OS lines were concentrated to 2 × 10^4^ cells/40 µL of the appropriate medium and placed onto 10-well hydrophobic slides (Thermo Scientific, Waltham, MA, USA) for a 24 h incubation. After that, the culture medium was replaced, and the cells were incubated with selected concentrations of the tested compounds and their combinations (Table 2) for 24 h. The cells on the hydrophobic slides were fixed with 1% paraformaldehyde solution (POCH, Gliwice, Poland), and TUNEL staining with ApopTag^®^ Peroxidase An In Situ Apoptosis Detection Kit (Merck Millipore, Darmstadt, Germany) was performed. The nuclei were stained with 1% hematoxylin solution (Sigma, Germany). In the last step of the assay, the slides were immersed in 70% ethanol (Stanlab, Warszawa, Poland) for 30 s, then in xylene (Stanlab, Poland) for 30 s, and covered with a cover slip and slide adhesive (Thermo Scientific, Waltham, MA, USA).

**Figure 1:**
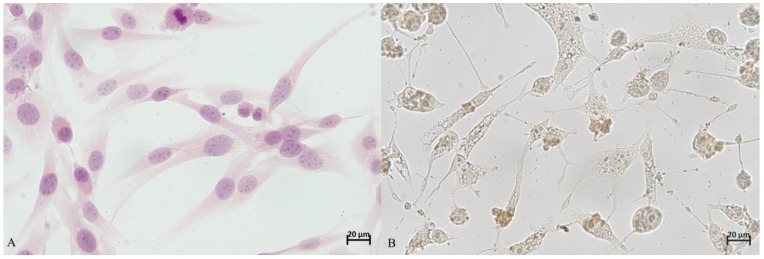
The comparison of microscopic picture of non-apoptotic (**A**) and apoptotic (**B**) cell cultures used in this study (D-17), 40×.

The percentage of apoptotic cells was determined in five fields of vision at a magnification of 40× under an optical microscope, Olympus BX53 (Olympus, Tokyo, Japan). In the first step all cells visible in the field of vision were counted (100%). Next, the number of apoptotic cells was estimated, and their percentage computed. The procedure was repeated in five fields of vision for every well (Figure 1).

The observation results were expressed as a mean of five evaluated fields of vision. This assessment was performed by two independent researchers with considerable experience in evaluation of immunohistochemical reactions.

### 2.4. Statistical Analysis

The statistical analysis of the data was performed with StatisticaPL 10.0 software (StatSoft, Kraków, Poland). The normality of results was verified with a Shapiro–Wilk W test. Subsequently, the values estimated for particular compounds and their combinations were compared with a Kruskal–Wallis test. A Mann–Whitney U test was used to compare the results for canine and human cell lines. The correlation analysis was performed with Spearman’s correlation test. The significance level was established at *p* < 0.05.

## 3. Results

### 3.1. Cell Viability

The statistical analysis consisted of the comparison of meloxicam and risedronate sodium per se cytotoxicity against human and canine OS cell lines. The EC_50_ value was computed for both compounds’ activity (Table 3).

The non-steroidal, anti-inflammatory drug meloxicam showed higher cytotoxicity against the canine OS cell line than the human. At its highest concentration (300 µg/mL), it narrowed the viability of the cells to 1.92 ± 4.89% for D-17 and 22.37 ± 4.63% for human OS (U-2 OS). The concentration of 100 µg/mL of the aforementioned compound caused low cytotoxicity against the canine OS and did not influence the variability in case of the human OS cell line (Table 4). The comparison of both meloxicam cytotoxic effects (concentrations 300 and 100 µg/mL) proved statistically significant differences (*p* < 0.05).

We decided to investigate risedronate sodium, the third-generation bisphosphonate, as it is quite a new drug that has not been frequently used in the therapy of dogs or humans. The highest concentration of risedronate sodium (300 µg/mL) narrowed the cell variability of human and canine OS to 14.84 ± 2.36% and 29.1 ± 2.17%, respectively. The cytotoxic effect of risedronate sodium was stronger against human OS. Risedronate sodium cytotoxicity compared in two concentrations (100 and 300 µg/mL) showed the statistically significant differences only in the case of human OS (*p* < 0.05). Simultaneously, the comparison of cytotoxic effect between concentrations of 30 and 300 µg/mL proved statistically significant differences in both cases (human and canine OS) (*p* < 0.05). The administration of risedronate sodium in doses of 100 µg/mL caused a moderate cytotoxic effect in both investigated cell lines (D-17, U-2 OS), around 50% in comparison to the negative control (Table 4).

**Figure 2:**
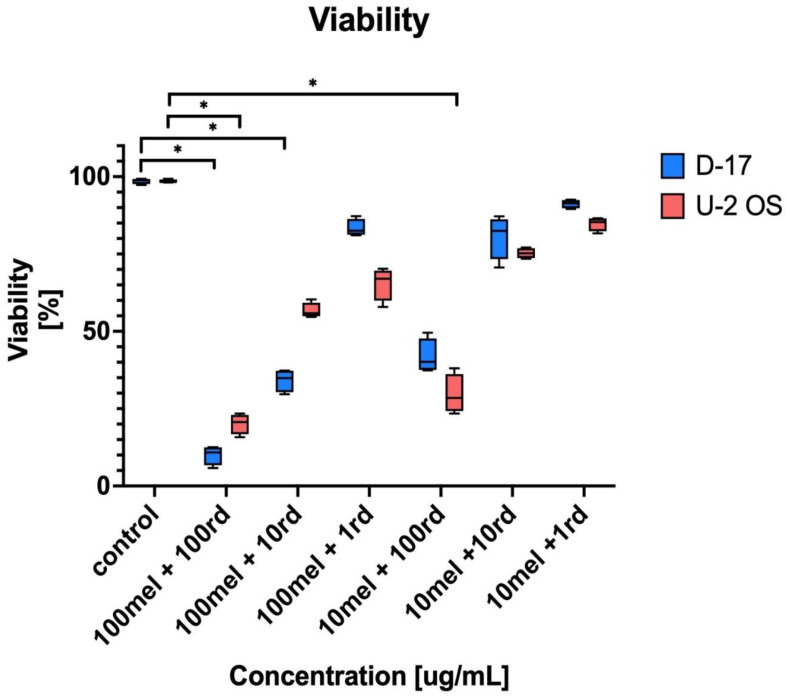
Cell viability for human and canine OS cell lines (U-2 OS and D-17) treated with combined risedronate sodium (rd) and meloxicam (mel) administration (* *p* < 0.05). The statistical analysis proved that the cytotoxic effect of 100 mel + 100 rd and 100 mel + 10 rd combinations against D-17 were, statistically, significantly different to the negative control (control). In the case of U-2 OS, the cytotoxicity of 100 mel + 100 rd and 10 mel + 100 rd combinations proved statistically significant differences compared to the negative control.

The statistical analysis of risedronate sodium and meloxicam combined administration effect showed subsequent results (Figure 2).

The combination of risedronate sodium and meloxicam in doses of 100 µg/mL (100 mel/100 rd) compared with negative control showed statistically significant differences in the viability of human and canine OS cell lines, U-2 OS: 20.14 ± 3.27% and D-17: 10.01 ± 3.13% (negative control 98.51 ± 0.55%), *p* < 0.05, respectively. The cytotoxic effect was stronger in canine OS (D-17).

Moreover, the combination of meloxicam, in doses of 100 µg/mL, and risedronate sodium, in doses of 10 µg/mL, showed lesser viability of canine OS than the negative control (statistically significant), D-17: 34.18 ± 3.75% and 98.58 ± 0.91%, *p* < 0.05. Finally, 10 µg/mL of meloxicam and 100 µg/mL risedronate sodium combination compared to the negative control proved the statistically significant differences in the human OS cell line viability, U-2 OS: 29.62 ± 6.32% and 98.51 ± 0.55%, *p* < 0.05. The positive control (doxorubicin 0.5 µg/mL) proved cell viability at 20.89 ± 5.95% for canine OS and 9.14 ± 0.98% for human OS, respectively. The use of a ten-times lower dose of meloxicam, together with the same concentration of risedronate sodium, enhanced the cytotoxic effect against the investigated cell lines, and the viability of human (U-2 OS) was lower than canine (D-17) OS (29.62 ± 6.32% and 41.81 ± 5.58%, respectively).

### 3.2. Apoptosis

**Figure 3:**
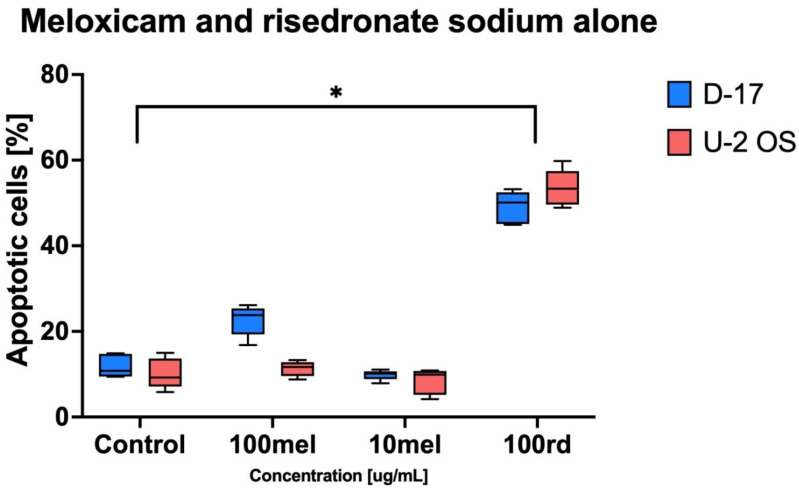
The apoptotic effect in canine (D-17) and human (U-2 OS) osteosarcoma triggered by risedronate sodium (rd) and meloxicam (mel) (* *p* < 0.05). The cytotoxic effect induced with 100 µg/mL of risedronate sodium proved statistically significant differences between both tested cell lines compared to the negative control (control). The results obtained with 10 and 100 µg/mL of meloxicam were not statistically significant.

The study demonstrated the strongest apoptosis induction activity (*p* < 0.05) against human OS (U-2 OS in the variant with risedronate sodium at 100 µg/mL. The percentage of programmed death cells was 53.5 ± 4.28%. The compound also showed high pro-apoptotic activity against canine OS (D-17), which was estimated at 49.06 ± 3.77%. Meloxicam alone did not exert significant proapoptotic activity either in canine or human OS (Figure 3).

**Figure 4:**
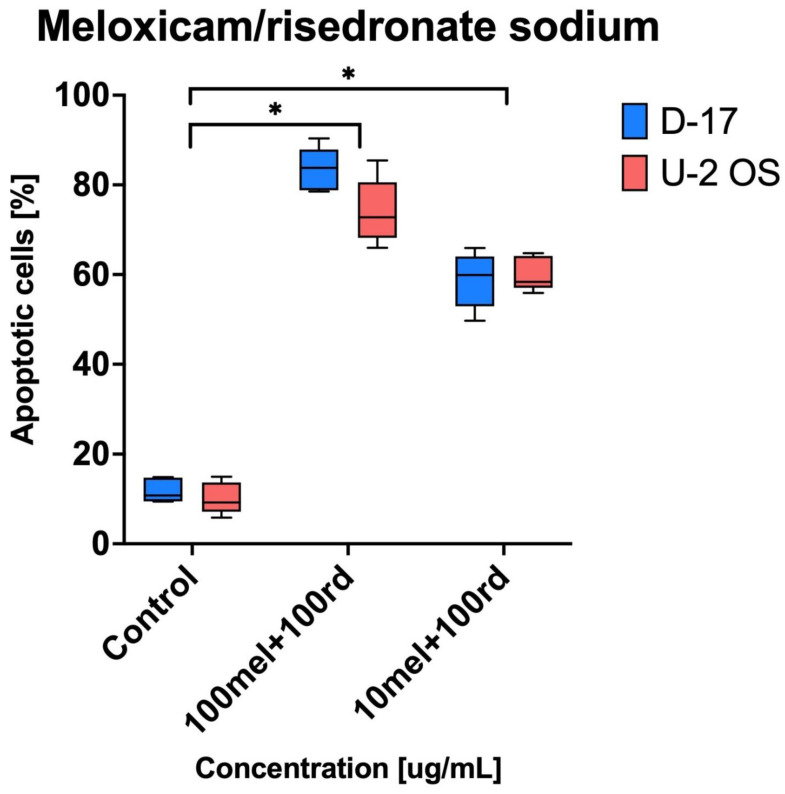
The pro-apoptotic effect on canine (D-17) and human (U-2 OS) osteosarcoma triggered with risedronate sodium/meloxicam combinations (* *p* < 0.05). The meloxicam and risedronate sodium combinations 100 mel + 100 rd and 10 mel + 100 rd compared to the negative (control) showed the statistically significant differences. The cytotoxic effect caused by 100 mel + 100 rd was stronger than induced by 10 mel + 100 rd.

Figure 4 shows a combined, significant effect of meloxicam (100 and 10 µg/mL) and risedronate sodium (100 µg/mL) on apoptosis in the canine and human OS cell lines. The exposition of the cell lines to risedronate sodium (100 µg/mL) and meloxicam (100 µg/mL) resulted in the apoptosis rate of 83.43 ± 4.88 and 74.08 ± 7.29%, respectively. Additionally, for the ten-times lower concentration of meloxicam (10 µg/mL) and the same concentration of risedronate sodium, the apoptosis was seen in around 60% of human and canine OS cells (60.17 ± 3.81 and 58.76 ± 6.17).

## 4. Discussion

Meloxicam is one of the most common non-steroidal anti-inflammatory drugs (NSAID) used in veterinary medicine, while in humans its application is less popular. It is often assumed that this substance exhibits anticancer activity against different types of tumors, both in humans and animals, as confirmed for hepatocellular carcinoma (HCC), non-small cell lung cancer, bladder cancer, colon cancer, or OS [25,26,27,28,29,30].

Studies assessing the use of meloxicam against cells from the established canine (D-17) and human (U-2 OS) OS lines demonstrated significantly higher viability and EC_50_ of human than canine OS cells (Table 3). This finding confirmed that canine OS cells are less resistant to the cytotoxic activity of meloxicam (*p* < 0.05) than humans OS cell lines (Table 4). This phenomenon may be due to the moderate-selective inhibition of COX-2 in humans. Human OS cell lines (HOS and U2-OS) exhibit low levels of COX-2, which is interpreted as the main cause of the apoptotic effect of meloxicam [26]. The different effectiveness of cyclooxygenase II blockers in dogs such as meloxicam, may be an explanation for the differences in cytotoxicity between the human and canine OS cell lines we observed.

Additionally, the concentration-dependent cytotoxic activity of meloxicam could be observed in both canine and human OS cell lines. In this study, the highest cytotoxic activity was found for the highest concentrations, i.e., 100–300 µg/mL, while the lowest tested concentrations induced only a slight stimulation of cellular proliferation. In both tested cell lines, a negative correlation between viability and meloxicam concentration (*p* < 0.05, r = 42) was observed. In a study on another line of canine OS cells (D-17), Wolfesberger et al. [25] reported a similar response in high doses of meloxicam (0–600 µM/mL) after a 72 h exposure. Simultaneously, low doses of meloxicam (10 µM/mL) resulted in slight stimulation of cellular proliferation, which was also confirmed in our studies. The cited authors reported EC_50_ values of 215.9 µM/mL, which were lower than our findings. The most probable explanation is the different types of active substances and diluents used in the two studies. That is, Wolfesberger et al. [25] used DMSO as diluent, which at specific concentrations may exhibit potent cytotoxic activity; however, data on final concentration of DMSO were not provided.

Naruse et al. [16] studied cells from the established human U-2 OS cell line treated with meloxicam at 10, 50, and 100 µM/mL. They concluded that meloxicam at the above-mentioned concentrations did not exhibit any cytotoxic effect in the tested cell line. In our study, we observed a slight increase in cell proliferation at the above-mentioned concentrations. The difference was minute and may have resulted from a methodological error.

We also investigated a bisphosphonate (risedronate sodium) combined with meloxicam. Even though the literature contains some reports on cytotoxic activity of older bisphosphonates [22], risedronate sodium is a new representative of this group (third generation) and has, so far, rarely been taken into account in in vitro studies. Bisphosphonates are used in the treatment of, e.g., metabolic bone tissue disorders, but their cytotoxicity predisposes them to the treatment of neoplasms, like myeloma multiplex or osteosarcoma. These properties also sparked the interest of other researchers [21,22,29,30]. After testing risedronate sodium activity against human and canine OS in a wide range of concentrations, we found that its cytotoxicity was concentration-dependent (*p* < 0.05). For example, in D-17 and U-2 OS treated with 100, 10, and 1 µg/mL risedronate sodium solution, the viability equaled 53.38 ± 1.46 and 49.56 ± 0.7%; 97.08 ± 3.32 and 74.92 ± 4.01%; and 102.67 ± 3.56 and 94.56 ± 3.52%, respectively. The negative correlation between the concentration on the tested substance and cell viability was proved in both canine (*p* < 0.05, r = 0.42) and human OS (*p* < 0.05, r = 48). The analysis of alive cell percentage and EC_50_ (D-17; 144.83 ± 6.22 µg/mL and U-2 OS; 98.1 ± 5.4 µg/mL) indicated a higher susceptibility of human than canine OS cells to the cytotoxicity of risedronate sodium (*p* < 0.05) (Table 3). Similar studies on risedronate sodium activity [at 0.1, 1 and 10 µM/mL] against human and canine OS cell lines were carried out by Xin et al. [20] and demonstrated no significant influence of risedronate sodium at those doses on the examined cell lines. Our findings were similar for the lower concentrations, but at 100 µg/mL a clear and strong cytotoxic effect was visible (Table 4). While comparing our results with those of Poirier et al. [22], who used one of the third-generation bisphosphonates (zelodronate sodium), it is important to point out that its strong cytotoxicity was proven against canine OS (D-17 and Abrams) and human OS (MG-63 and SAOS-2). This property was also concentration-dependent, and the cytotoxic effect was visible for higher concentrations of the investigated compound. The latter assumption is similar to the results of our study. The cytotoxic activity against human and canine OS cell lines was confirmed not only for the third but also the second generation of bisphosphonates (aledronate sodium) by Farese et al. [21] and Poirier et al. [22]. The findings of those authors were concurrent with our results on the concentration-dependent activity of bisphosphonates observed in OS cell cultures. In our case, the synergistic activity of meloxicam and risedronate sodium was evident and was the highest for the combination with 100 µg/mL meloxicam (Table 3). Moreover, the cytotoxic effect of this drug combination was greater in the canine OS cell line than the human (*p* < 0.05). In summary, among all tested combinations of meloxicam and risedronate sodium, the highest cytotoxic activity was detected for 100 µg/mL risedronate sodium + 100µg/mL meloxicam (*p* < 0.05) and, in comparison with negative control (non-treated cells), the statistically significant differences in the viability of human and canine OS cell line were proved (Figure 2). The cytotoxic effect was stronger against canine OS (D-17).

The main question is if the aforementioned effect can be classified as additive or synergic activity of the combined drugs. Due to the different mechanism of action and target of risedronate sodium and meloxicam, the increase in cytotoxic effect in the chosen OS cell lines can be ascribed to synergy (Table 4). The administration of meloxicam per se in a dose of 100 µg/mL induced low cytotoxicity against the canine OS cell line only and the viability equal 83.3% (±3.37). On the other hand, risedronate sodium, in the same dose, caused a cytotoxic effect in 50% of both human and canine OS cell lines compared to the negative control. The combined use of both compounds in doses of 100 µg/mL resulted in a decrease in viability to 10.01 % ± 3.13 (D-17) and 20.14% ± 3.27. These values have shown the statistically significant differences (*p* < 0.05) and it is possible to conclude that combined use of meloxicam and risedronate sodium in comparison to both negative control and to separate administration can be classified as synergy. In the case of apoptosis, it was clearly visible that the combination of risedronate sodium and meloxicam induced apoptosis in human and canine OS cell lines (Figure 4). The strongest apoptotic effect was observed for 100 µg/mL of risedronate sodium combined with 100 µg/mL of meloxicam (D-17: 83.43% ± 4.88 and U-2 OS: 74.08% ± 7.29). The statistical analysis proved the significant differences not only in combination in doses of 100 µg/mL, but also in combination of meloxicam and risedronate sodium in doses of 10 µg/mL and 100 µg/mL. On the other hand, meloxicam alone is not a strong inducer of apoptosis (D-17: 22.63 ± 3.60% and U-2 OS: 11.29 ± 1.76%; Figure 2), as also reported by Wolfesberger et al. [24] in their study on D-17, where the percentage of apoptotic cells in the group treated with 200 µM/mL meloxicam was 12.26 ± 3.35%, and with 400 µM/mL was 20.16 ± 3.83%. We recorded similar values in our study, where the percentage of apoptotic cells reached 22.63 ± 3.6% in the sample treated with 100 µg/mL of meloxicam. Contrary to that, Naruse et al. [16] reported a lack of apoptosis induction by meloxicam at 100 µM/mL in human OS (U-2 OS and HOS) cell lines. Our study yielded similar results for higher concentrations of meloxicam and U-2 OS. Our experiments demonstrated strong pro-apoptotic properties of risedronate sodium at the tested concentrations in both human and canine OS cell line. Murayama et al. [18] confirmed the pro-apoptotic activity of risedronate sodium in OS cell line (LM-8), and also proved its concentration-dependent nature. Finally, it is important to keep in mind that not only risedronate sodium, but also zelodronate sodium (the third generation) and aledronate sodium (the second-generation bisphosphonate), can induce a strong pro-apoptotic response in OS cell lines [22]. Our study demonstrated the statistically significant effect of meloxicam and risedronate sodium on apoptosis in the investigated canine and human OS cell lines. Additionally, for the ten-times lower concentration of meloxicam (10 µg/mL) and the same concentration of risedronate sodium, the apoptosis was in 60% of human and canine OS cells. All mentioned values showed statistically significant differences. Moreover, we used other cell lines, which may potentially exhibit higher susceptibility to the tested substances. In our study, the cell line exposure to the combinations of meloxicam and risedronate sodium demonstrated that the drugs used together provide a stronger cytotoxic effect (Figure 2) than risedronate sodium alone, and significant cytotoxicity of meloxicam alone was not proven either (Table 4).

Even though the in vitro studies showed the statistically significant difference between the use of risedronate sodium and meloxicam per se and in combinations against both human and canine OS cell lines, we must stress that the potential concentration of the investigated compound in tissues and plasma can be narrowed to values lower than in culture media. Therefore, the cytotoxic effect observed in vitro may or may not be transferable to living tissues. The concentration of 100 µg/mL cannot be achieved in plasma and tissue in vivo (meloxicam maximal concentration is 1 µg/mL).

## 5. Conclusions

The study indicated a synergetic effect of risedronate sodium and meloxicam used in combination. Despite the fact that meloxicam alone did not exhibit important pro-apoptotic effects at the tested concentrations, its combinations with the second investigated compound enhanced the capability of risedronate sodium to induce apoptosis in the investigated neoplasm cell lines.

## Figures and Tables

**Table 1:** Drug combinations used in the experiment: mel-meloxicam + rd-risedronate sodium.

Combinations [µg/mL]
100 mel + 100 rd
100 mel + 10 rd
100 mel + 1 rd
10 mel + 100 rd
10 mel + 10 rd
10 mel + 1 rd

**Table 2:** Drug combinations used in TUNEL assay: mel-meloxicam, +rd-risedronate sodium.

Combinations [µg/mL]
100 mel + 100 rd
10 mel + 100 rd

**Table 3:** EC_50_ values computed for risedronate sodium and meloxicam cytotoxicity. Similar concentration of both drugs caused a comparable effect against the canine OS cell line (D-17). In case of human OS (U-2 OS), the stronger cytotoxic effect of risedronate sodium was proved. The EC_50_ values for meloxicam in both cell lines were, statistically, significantly different (* *p* < 0.05).

	EC_50_		SD	
Cell Line	D-17	U-2 OS	D-17	U2-OS
risedronate sodium	144.83 µg/mL	98.1 µg/mL	±6.22	±5.4
meloxicam	149.9 µg/mL *	234.02 µg/mL *	±9.17	±5.96

**Table 4:** The viability of tested compounds per se and in combinations (* *p* < 0.05). The cytotoxic effect of combined administration of meloxicam (mel) and risedronate sodium (rd) in doses of 100 µg/mL compared with the negative control (control) showed statistically significant differences. The human OS cell line (U-2 OS) was more resistant to both compounds than the canine OS cell line (D-17).

Cell Line	Compound	Viability	SD
D-17	control	98.58	0.91
	100 mel	83.3	3.37
	100 rd	53.12	1.46
	100 mel + 100 rd *	10.01	3.13
U-2 OS	control	98.51	0.55
	100 mel	100	4.25
	100 rd	49.57	0.7
	100 mel + 100 rd *	20.14	3.27

## Data Availability

Not applicable.
